# The roles of the Na^+^/K^+^‐ATPase, NKCC, and K^+^ channels in regulating local sweating and cutaneous blood flow during exercise in humans in vivo

**DOI:** 10.14814/phy2.13024

**Published:** 2016-11-23

**Authors:** Jeffrey C. Louie, Naoto Fujii, Robert D. Meade, Glen P. Kenny

**Affiliations:** ^1^ Human and Environmental Physiology Research UnitSchool of Human KineticsUniversity of OttawaOttawaONCanada

**Keywords:** Exercise, heat loss, K^+^ channels, microcirculation, Na^+^/K^+^‐ATPase, NKCC, sweat gland

## Abstract

Na^+^/K^+^‐ATPase has been shown to regulate the sweating and cutaneous vascular responses during exercise; however, similar studies have not been conducted to assess the roles of the Na‐K‐2Cl co‐transporter (NKCC) and K^+^ channels. Additionally, it remains to be determined if these mechanisms underpinning the heat loss responses differ with exercise intensity. Eleven young (24 ± 4 years) males performed three 30‐min semirecumbent cycling bouts at low (30% VO_2peak_), moderate (50% VO_2peak_), and high (70% VO_2peak_) intensity, respectively, each separated by 20‐min recovery periods. Using intradermal microdialysis, four forearm skin sites were continuously perfused with either: (1) lactated Ringer solution (Control); (2) 6 mmol·L^−1^ ouabain (Na^+^/K^+^‐ATPase inhibitor); (3) 10 mmol·L^−1^ bumetanide (NKCC inhibitor); or (4) 50 mmol·L^−1^ BaCl_2_ (nonspecific K^+^ channel inhibitor); sites at which we assessed local sweat rate (LSR) and cutaneous vascular conductance (CVC). Inhibition of Na^+^/K^+^‐ATPase attenuated LSR compared to Control during the moderate and high‐intensity exercise bouts (both *P* ˂ 0.01), whereas attenuations with NKCC and K^+^ channel inhibition were only apparent during the high‐intensity exercise bout (both *P* ≤ 0.05). Na^+^/K^+^‐ATPase inhibition augmented CVC during all exercise intensities (all *P* ˂ 0.01), whereas CVC was greater with NKCC inhibition during the low‐intensity exercise only (*P* ˂ 0.01) and attenuated with K^+^ channel inhibition during the moderate and high‐intensity exercise conditions (both *P* ˂ 0.01). We show that Na^+^/K^+^‐ATPase, NKCC and K^+^ channels all contribute to the regulation of sweating and cutaneous blood flow but their influence is dependent on the intensity of dynamic exercise.

## Introduction

The physiological mechanisms regulating the heat loss responses of sweating and cutaneous vasodilatation during exercise have yet to be fully elucidated. It is generally accepted that the production of sweat is facilitated by the transport of various ions (e.g., Na^+^, K^+^, Cl^−^) to establish a series of electrochemical gradients across the basolateral and luminal membranes on the sweat gland (Sato et al. 1989). The process of sweat production has been explained by the Na‐K‐2Cl co‐transporter (NKCC) model (Quinton 1983; Sato et al. 1989; Saga 2002), which highlights the involvement of various membrane transport proteins including the sodium pump (Na^+^/K^+^‐ATPase), NKCC, and K^+^ channels. Indeed, Na^+^/K^+^‐ATPase has been demonstrated to regulate sweating in humans during exercise, evidenced by large attenuations in sweat rate following local administration of the Na^+^/K^+^‐ATPase inhibitor ouabain (Sato and Dobson 1969; Sato et al. 1969; Louie et al. 2016). In contrast, despite the NKCC and K^+^ channels being implicated in the production of sweat in vitro (Sato and Sato 1987; Reddy and Quinton 1991, 1999; Suzuki et al. 1991; Samman et al. 1993; Sato et al. 1993; Toyomoto et al. 1997; Bovell et al. 2008), it is unknown if these findings can be extended to the responses in humans in vivo.

In our recent study, we demonstrated that Na^+^/K^+^‐ATPase inhibition augmented cutaneous vasodilatation during exercise in the heat (Louie et al. 2016). We had determined that the augmented cutaneous vasodilatory response seen with Na^+^/K^+^‐ATPase inhibition was nitric oxide‐dependent (Louie et al. 2016). Furthermore, although studies have demonstrated that NKCC inhibition results in relaxation of vascular smooth muscle (Liguori et al. 1999; Garg et al. 2007; Orlov 2007) and K^+^ channel inhibition typically causes an attenuated cutaneous vascular response (Hojs et al. 2009; Brunt and Minson 2012; Kutz et al. 2015), none have addressed their roles in cutaneous vasodilatation in humans during exercise.

It was recently observed that key modulators of the sweating and cutaneous vasodilatory responses, such as those associated with the influence of nitric oxide synthase, varied as a function of exercise intensity (Fujii et al. 2014; Meade et al. 2015). Although the underlying mechanism(s) to explain these variations remain unknown, there exist several factors that may affect the regulation of sweating and cutaneous vasodilatation that have been demonstrated to increase in production or activation in parallel with the level of exercise intensity, such as those associated with aldosterone and vasopressin (Montain et al. 1997), heat‐shock proteins (i.e., HSP70) (Milne and Noble 2002), endothelin‐1 (Maeda et al. 1994), and oxidative stress (Goto et al. 2003). However, given the fundamental nature of the downstream regulators Na^+^/K^+^‐ATPase, NKCC, and K^+^ channels, and their likely contribution to the regulation of the heat loss responses as hypothesized above, these regulators may also demonstrate similar intensity‐dependent responses as previously observed (Fujii et al. 2014; Meade et al. 2015).

The purpose of this study was to investigate the roles of the Na^+^/K^+^‐ATPase, NKCC, and K^+^ channels in regulating the local sweating and cutaneous vasodilatory responses during exercise. Moreover, we sought to determine whether the contributions to the heat loss responses of each of these transporters varied depending on the level of exercise intensity and therefore rate of metabolic heat production. We hypothesize that inhibition of the Na^+^/K^+^‐ATPase, NKCC, and K^+^ channels will result in attenuations in local sweat rate, whereas Na^+^/K^+^‐ATPase and NKCC inhibition will augment cutaneous vasodilatation and K^+^ channel inhibition will attenuate this response. Further, we hypothesized that the contributions of each transporter would be attenuated with greater levels of exercise intensity.

## Methods

### Ethical approval

This study obtained approval from the University of Ottawa Health Sciences Ethics Board and conformed to the guidelines set forth by the *Declaration of Helsinki*. Written and informed consent were acquired prior to involvement in the study.

### Participants

Eleven young, healthy, and physically active (2–5 days·week^−1^ of structured physical activity; ≥30 min·day^−1^) males were recruited to participate in this study. Participants were normotensive, nonsmoking, nonheat acclimatized, and were excluded if they had any history of cardiovascular, respiratory, and/or metabolic diseases. Participants’ characteristics (mean ± standard deviation) were as follows: age, 24 ± 4 years; height, 1.78 ± 0.07 m; mass, 75.7 ± 10.5 kg; body surface area, 1.9 ± 0.2 m^2^; body fat percentage, 14 ± 4%; peak rate of oxygen consumption (VO_2peak_), 48 ± 5 mL O_2_·kg^−1^·min^−1^.

### Experimental procedures

#### Preliminary testing session

Participants were required to undergo a preliminary testing session. During this time, their anthropometric and VO_2peak_ data were collected for screening purposes. Participants were asked to refrain from food (≥2 h prior to the session), alcohol, caffeine, high‐intensity exercise (≥12 h) and over‐the‐counter and/or prescriptions medications (including supplements such as vitamins and minerals) (≥24 h) prior to this session. We measured body height and mass using a stadiometer (Detecto, model 2391, Webb City, MO) and digital high‐performance weighing terminal (model CBU150X, Mettler Toledo Inc., Mississauga, ON, Canada), respectively, and these measurements were subsequently used to determine body surface area (Du Bois and Du Bois 1989). The hydrostatic weighing technique was utilized to determine body density, and from this, body composition was estimated (Siri 1956). We assessed VO_2peak_ using an incremental exercise protocol until exhaustion on a semi‐recumbent cycle ergometer (Corival Recumbent, Lode, Groningen, Netherlands). The initial workload was set to 100 W, increasing by 20 W·min^−1^. Participants were instructed to maintain a pedaling cadence of 60–90 revolutions·min^−1^ and the test concluded when the participant reached volitional fatigue or could not maintain ≥50 revolutions·min^−1^. Expired air was concomitantly assessed using an automated indirect calorimetry system (MCD Medgraphics Ultima Series, MGC Diagnostics, MN) and VO_2peak_ was taken as the greatest average oxygen uptake over a period of 30 sec.

#### Experimental testing session: intradermal microdialysis fiber placement

On a separate day (≥48 h from the preliminary testing session), participants underwent the experimental testing session. Participants were asked to refrain from the same items outlined above for the preliminary testing sessions prior to arriving to the laboratory on this day. Participants were instructed to adequately hydrate prior to the experimental testing session by consuming ≥500 mL of water the night prior and roughly 2 h before arriving to the laboratory. Urine samples were collected to assess hydration via urine specific gravity (1.007 ± 0.002) (Sawka et al. 2007). Following a measurement of nude body mass, participants were seated semirecumbently in a thermoneutral room (25°C). Four intradermal microdialysis fibers (30 kDa cutoff; MD2000, Bioanalytical Systems, West Lafayette, IN) were instrumented in aseptic conditions to the dermal layer of skin on the dorsal side of the left forearm. This was accomplished using a 25‐gage needle inserted into the nonanesthetized skin which traveled ~2.5 cm before exiting. Following needle placement, the microdialysis fiber was threaded through the needle's lumen. By carefully withdrawing the needle, the 10 mm semipermeable membrane of the microdialysis fiber was situated in the forearm skin. The fiber was then secured in place to the skin using surgical tape. This process was repeated for placement of the remaining three fibers, each being placed ≥4 cm aside from one another.

#### Experimental testing session: exercise protocol

After placement of the intradermal microdialysis fibers, participants were directed to a thermal chamber located in an adjacent room (Can‐Trol Environmental Systems, Markham, ON, Canada) regulated to 25°C and 20% relative humidity and seated on a semirecumbent cycle ergometer. They remained in this seated position for the remainder of the experimental testing session. In a counterbalanced manner, the microdialysis fibers were perfused with one of the following pharmacological agent solutions: (1) lactated Ringer solution (Control); (2) 6 mmol·L^−1^ ouabain (Ouabain; Sigma‐Aldrich, St Louis, MO), Na^+^/K^+^‐ATPase inhibitor; (3) 10 mmol·L^−1^ bumetanide (Bumetanide; Cayman Chemical, Ann Arbor, MI), NKCC inhibitor; or (4) 50 mmol·L^−1^ BaCl_2_ (BaCl_2_; Sigma‐Aldrich), nonspecific K^+^ channel inhibitor at a rate of 4 μL·min^−1^ for the remainder of the trial using a microinfusion pump (Model 400, CMA Microdialysis, Solna, Sweden). A habituation period (≥60 min) of drug perfusion prior to baseline data collection was undertaken at all four skin sites to ensure a complete blockade was established. The concentration of ouabain (i.e., 6 mmol·L^−1^) was chosen based on a previously conducted study in our lab that used this agent (Louie et al. 2016). Given that the pharmacological agents bumetanide and BaCl_2_ had not been utilized with the intradermal microdialysis technique as NKCC and nonspecific K^+^ channel inhibitors, respectively, it was necessary to conduct pilot work to determine the appropriate concentrations of these drugs. Insight was gleaned from previous studies utilizing 10 mmol·L^−1^ bumetanide (Garg et al. 2007) and 2 μmol·L^−1^ to 10 mmol·L^−1^ BaCl_2_ (Sato and Sato 1987; Nelson and Quayle 1995; Brunt et al. 2013). Moreover, although Brunt et al. (2013) had previously administered 100 μmol·L^−1^ BaCl_2_ via intradermal microdialysis, they chose this lower concentration to inhibit only the inwardly rectifying (K_IR_) and ATP‐sensitive (K_ATP_) K^+^ channels, whereas other K^+^ channels, such as the calcium‐activated (K_Ca_) and voltage‐gated (K_V_) K^+^ channels likely remained active. The pilot work for this study involved the placement of microdialysis fibers, as previously described, perfused with lactated Ringer solution (Control), or various low, medium, or high concentrations of either bumetanide or BaCl_2_ (i.e., separate trials). To elicit increases in sweating and cutaneous vasodilatation, participants exercised at a constant rate of metabolic heat production of 500 W until a steady state in the responses was attained (approximately 30–45 min). For bumetanide, it was determined that 10 mmol·L^−1^ (dissolved in lactated ringers and 20 mmol·L^−1^ NaOH) was the highest concentration able to be made and resulted in the largest attenuation in local sweat rate (a decrease of ~1.05 mg·min^−1^·cm^−2^ relative to Control) at the end of the exercise bout. With regard to BaCl_2_, it was observed that 50 mmol·L^−1^ BaCl_2_ attenuated the end‐exercise cutaneous vasodilatory response to a similar extent as the administration of 100 mmol·L^−1^ of BaCl_2_ (a reduction of ~33% of maximal cutaneous vascular conductance (CVC_max_) relative to Control), both of which were greater than 5 mmol·L^−1^ BaCl_2_ (a decrease of ~6% CVC_max_ relative to Control).

Following the habituation period of the experimental testing session, the exercise protocol consisted of a 5 min baseline period. Participants then performed three successive 30‐min bouts of exercise at a low (30% VO_2peak_), moderate (50% VO_2peak_), and high (70% VO_2peak_) intensity (equivalent to a rate of metabolic heat production of 316 ± 36, 497 ± 45, and 715 ± 70 W, respectively; an equivalent external workload of 60 ± 8, 111 ± 9, and 149 ± 13 W, respectively). Each exercise bout was followed by a 20‐min recovery period. After completion of the last recovery period, the final stage of the experimental protocol was employed to elicit maximal cutaneous vasodilatation. All fibers were perfused with 50 mmol·L^−1^ sodium nitroprusside (Sigma‐Aldrich) at a rate of 6 μL·min^−1^ for the remainder of the ~20 min period until a stable 2‐min plateau in cutaneous blood flow measurements was established. Blood pressure was measured after cessation of this final period and was used to calculate maximal cutaneous vascular conductance (CVC_max_). Lastly, the fibers were removed from the forearm skin.

### Measurements

Local forearm sweat rate (LSR) was measured with the ventilated capsule technique in which each skin site was designated a sweat capsule covering a surface area of 1.1 cm^2^, specially designed for use with intradermal microdialysis to account for the diffusion distance of the pharmacological agents (Meade et al. 2016). These capsules were placed directly over the semipermeable membrane of the fiber and were secured to the skin with adhesive rings and topical glue (Collodion HV, Mavidon Medical Products, Lake Worth, FL). Long vinyl tubes connected each compressed anhydrous air tank to a flow rate monitor (Omega FMA‐A2307, Omega Engineering, Stamford, CT), which was then connected to each capsule, and subsequently to a capacitance hygrometer (model HMT333, Vaisala, Helsinki, Finland). Moreover, the aforementioned equipment was located in the thermal chamber to ensure internal gas temperatures were equilibrated to near room temperature (25°C). The anhydrous air was delivered at a flow rate of 0.2 L·min^−1^. Every 5 sec LSR was calculated using the difference in water content between the influent and effluent air, multiplied by flow rate, and normalized for the skin surface area under the capsule (expressed as mg·min^−1^·cm^−2^).

Cutaneous red blood cell flux (expressed as perfusion units) was measured at a 32 Hz sampling rate with laser Doppler flowmetry (PeriFlux System 5000, Perimed, Stockholm, Sweden). An integrated laser Doppler flowmetry probe with a seven‐laser array (Model 413, Perimed) was housed in each specially designed sweat capsule, positioned on the skin directly over the semipermeable membrane of each fiber. This setup allowed for the simultaneous measurement of LSR and cutaneous red blood cell flux at each site. CVC was calculated as cutaneous red blood cell flux divided by mean arterial pressure and is presented as a percentage of the CVC_max_ acquired during the maximal skin blood flow protocol. Mean arterial pressure (calculated as diastolic pressure plus one‐third of systolic minus diastolic pressure [i.e., pulse pressure]) was determined at 5‐min intervals using manual auscultation with a validated mercury column sphygmomanometer (Baumanometer Standby Model, WA Baum Co, Copiague, NY).

Heart rate was measured continuously using a Polar coded WearLink and transmitter, Polar RS400 interface and Polar Trainer 5 software (Polar Electro, Kempele, Finland). Esophageal temperature was measured using a pediatric thermocouple probe (~2 mm diameter; Mon‐a‐therm; Mallinckrodt Medical, St Louis, MO) inserted ~40 cm past the entrance of the nostril. Mean skin temperature was measured using thermocouples (Concept Engineering, Old Saybrook, CT) at four skin sites and weighted to the following regional proportions: upper back, 30%; chest, 30%; quadriceps, 20%; and calf, 20%. Esophageal and skin temperature was collected at a sampling rate of 15 sec using a data acquisition module (Model 34970A; Agilent Technologies Canada, Mississauga, ON, Canada), displayed and recorded using LabVIEW software (National Instruments, Austin, TX). Using these data, mean body temperature was determined as (0.9 × core temperature) + (0.1 × skin temperature).

The rate of metabolic heat production was assessed as the difference between metabolic rate and external workload (Kenny and Jay 2013). Metabolic energy expenditure was measured using indirect calorimetry in which electrochemical gas analysers (AMETEK model S‐3A/1 and CD3A, Applied Electrochemistry, Pittsburgh, PA), calibrated using reference gas mixtures of known concentrations, were used to assess the oxygen and carbon dioxide of expired gas. Moreover, ventilation rate was measured with a turbine ventilometer that was calibrated using a 3 L syringe. In order to collect expired air, subjects wore a full face mask (Model 7600 V2, Hans‐Rudolph, Kansas City, MO), connected to a two‐way T‐shape non‐rebreathing valve (Model 2700, Hans‐Rudolph) and the respired gasses were averaged over periods of 30 sec.

### Data analysis

The values for LSR, CVC, heart rate, as well as esophageal, mean skin, and mean body temperature were obtained by averaging the measurements made over the last 5 min of each time period (i.e., exercise and recovery), whereas baseline values represent an average of the 5 min prior to the first exercise bout (i.e., low intensity). Blood pressure data were calculated as the average of the two measurements taken during 10‐min intervals. During the CVC_max_ period, the plateau was defined as the greatest CVC values averaged over 2 min. The differences (∆) in LSR and CVC from Control were calculated for the Ouabain, Bumetanide, and BaCl_2_ sites at the end of each exercise bout.

### Statistical analysis

Local forearm sweat rate and cutaneous vascular conductance were analyzed with separate two‐way repeated measures analyses of variance (ANOVAs) using the factors of time (7 levels: Baseline, Low Intensity Exercise, Recovery 1, Moderate Intensity Exercise, Recovery 2, High Intensity Exercise, and Recovery 3) and treatment site (4 levels: Control, Ouabain, Bumetanide, and BaCl_2_). Similarly, ∆LSR and ∆CVC from Control at the end of each exercise bout were analyzed using separate two‐way repeated measure ANOVAs with the factors of exercise period (3 levels: Low, Moderate, and High Intensity Exercise) and treatment site (3 levels: Ouabain, Bumetanide, and BaCl_2_). Esophageal, mean skin, and mean body temperatures, as well as mean arterial pressure and heart rate were analyzed using separate one‐way repeated measures ANOVAs with the factor of time (7 levels: Baseline, Low Intensity Exercise, Recovery 1, Moderate Intensity Exercise, Recovery 2, High Intensity Exercise, and Recovery 3). Absolute CVC_max_ (expressed in perfusion units ·mmHg^−1^) was analyzed with a one‐way repeated measures ANOVA using the factor of treatment site (4 levels: Control, Ouabain, Bumetanide, and BaCl_2_). *Post hoc* analyses were carried out using two‐tailed paired samples *t* tests adjusted for multiple comparisons using the Holm–Bonferroni procedure when a significant main effect was observed. Statistical analyses were completed using the software package SPSS 23.0 for Windows (IBM, Armonk, NY). For all analyses, *P* ≤ 0.05 was considered statistically significant. Values are presented as the mean ± 95% confidence intervals, unless otherwise indicated, calculated as 1.96 × standard error of the mean.

## Results

### Sweating response

**Figure 1:**
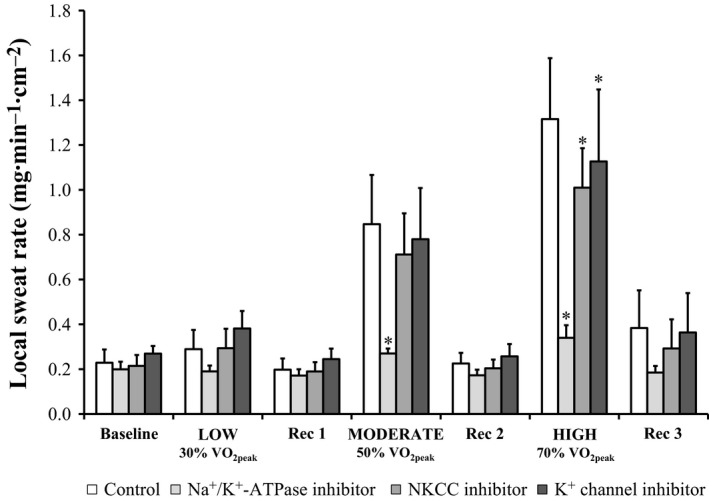
Local sweat rate at the end of each time period during intermittent 30‐min graded intensity exercise bouts separated by 20‐min recovery periods (n = 11). Four forearm skin sites were continuously perfused with: (1) lactated Ringer solution (Control, white); (2) 6 mmol·L^−1^ ouabain (Na^+^/K^+^‐ATPase inhibitor, light gray); (3) 10 mmol·L^−1^ bumetanide (NKCC inhibitor, medium gray); or (4) 50 mmol·L^−1^ BaCl_2_ (nonspecific K^+^ channel inhibitor, dark gray). Values are presented as mean ± 95% confidence interval. Baseline values represent the 5 min prior to the first exercise bout. All other values represent the final 5 min of the corresponding period. BL, baseline; LOW/MODERATE/HIGH, Low/Moderate/High intensity exercise bout; Rec, recovery period; VO_2peak_, peak rate of oxygen consumption. *Significantly different from Control; *P* ≤ 0.05.

**Figure 2:**
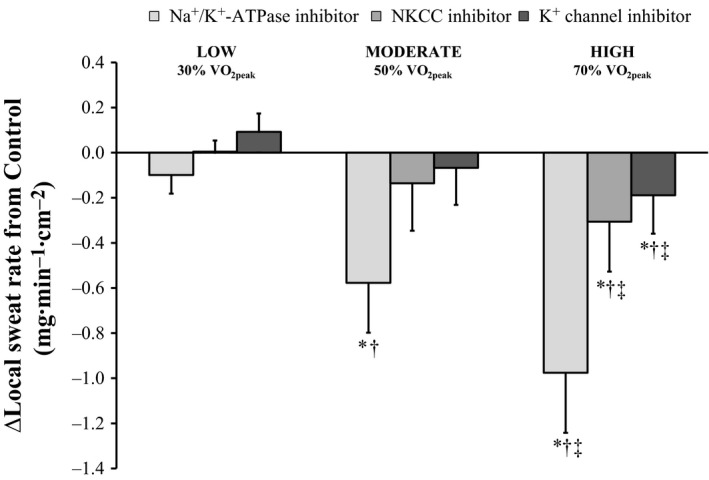
The difference (∆) in local sweat rate from Control at the end of each exercise bout (*n* = 11). Four forearm skin sites were continuously perfused with: (1) lactated Ringer solution (Control); (2) 6 mmol·L^−1^ ouabain (Na^+^/K^+^‐ATPase inhibitor, light gray); (3) 10 mmol·L^−1^ bumetanide (NKCC inhibitor, medium gray); or (4) 50 mmol·L^−1^ BaCl_2_ (nonspecific K^+^ channel inhibitor, dark gray). Values are presented as mean ± 95% confidence interval. Values represent the final 5 min of the corresponding period. LOW/MODERATE/HIGH, Low/Moderate/High intensity exercise bout; VO_2peak_, peak rate of oxygen consumption. *Significantly different from Control, ^†^significantly different from Ex 1, ^‡^Ex 3 significantly different from Ex 2; all *P* ≤ 0.05.

Local forearm sweat rate was similar to Control all treatment sites during Baseline (all *P* ≥ 0.17; Fig. 1) as well as at the end of the low‐intensity exercise bout (all *P* ≥ 0.06). At the end of the moderate exercise bout, LSR was attenuated at the Ouabain site (*P* ˂ 0.01) compared to Control (interaction of treatment site and time, *P* ˂ 0.01) but similar at the Bumetanide and BaCl_2_ sites (both *P* ≥ 0.24). LSR was reduced from Control during high‐intensity exercise at all treatment sites (all *P* ≤ 0.05). At the end of each recovery period, LSR was similar to Control at all treatment sites (all *P* ≥ 0.07). ∆LSR from Control at the Ouabain site was greater during both moderate and high‐intensity exercise compared to low (both *P* ˂ 0.01; Fig. 2), and greater during the high compared to the moderate intensity exercise bout (*P* ˂ 0.01). At the Bumetanide and BaCl_2_ sites, ∆LSR from Control was greater during high compared to both low and medium intensity exercise (all *P* ≤ 0.05).

### Cutaneous vascular response

**Figure 3:**
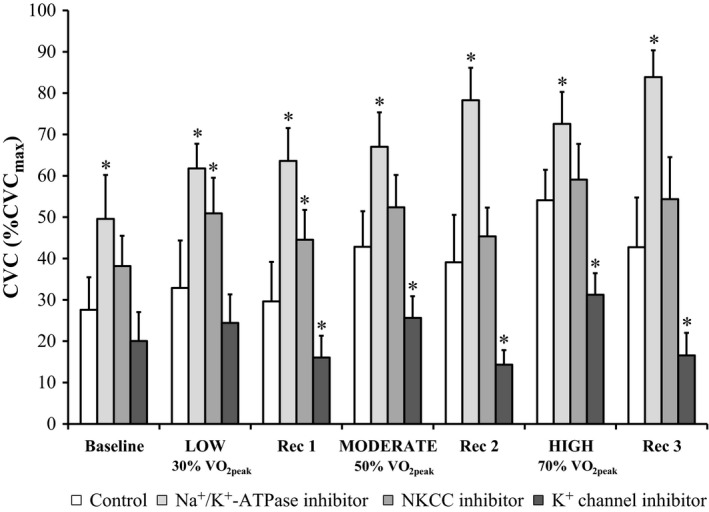
Cutaneous vascular conductance (CVC) at the end of each time period during intermittent 30‐min graded intensity exercise bouts separated by 20‐min recovery periods (*n* = 11). Four forearm skin sites were continuously perfused with: (1) lactated Ringer solution (Control, white); (2) 6 mmol·L^−1^ ouabain (Na^+^/K^+^‐ATPase inhibitor, light gray); (3) 10 mmol·L^−1^ bumetanide (NKCC inhibitor, medium gray); or (4) 50 mmol·L^−1^ BaCl_2_ (nonspecific K^+^ channel inhibitor, dark gray). Values are presented as mean ± 95% confidence interval. Baseline values represent the 5 min prior to the first exercise bout. All other values represent the final 5 min of the corresponding period. BL, baseline; LOW/MODERATE/HIGH, Low/Moderate/High intensity exercise bout; Rec, recovery period; VO_2peak_, peak rate of oxygen consumption. *Significantly different from Control; *P* ≤ 0.05.

**Figure 4:**
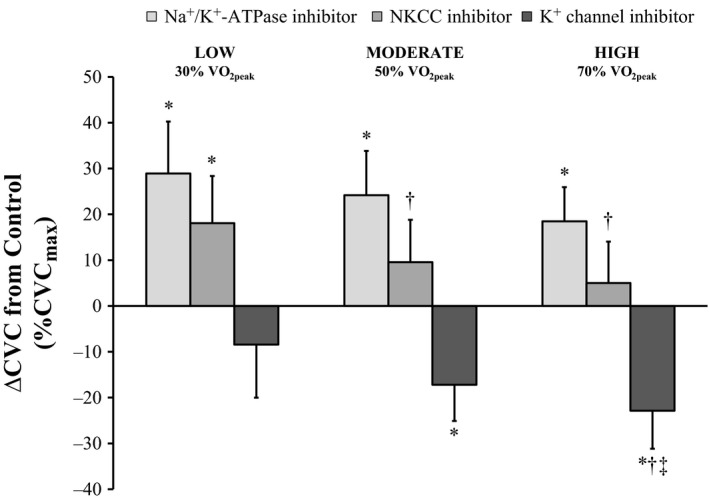
The difference (∆) in cutaneous vascular conductance (CVC) from Control at the end of each exercise bout (*n* = 11). Four forearm skin sites were continuously perfused with: (1) lactated Ringer solution (Control); (2) 6 mmol·L^−1^ ouabain (Na^+^/K^+^‐ATPase inhibitor, light gray); (3) 10 mmol·L^−1^ bumetanide (NKCC inhibitor, medium gray); or (4) 50 mmol·L^−1^ BaCl_2_ (nonspecific K^+^ channel inhibitor, dark gray). Values are presented as mean ± 95% confidence interval. Values represent the final 5 min of the corresponding period. LOW/MODERATE/HIGH, Low/Moderate/High intensity exercise bout; VO_2peak_, peak rate of oxygen consumption. *Control significantly different from treatment site, ^†^significantly different from Ex 1, ^‡^Ex 3 significantly different from Ex 2; all *P* ≤ 0.05.

Cutaneous vascular conductance was elevated at the Ouabain site at all time periods (all *P* ˂ 0.01; Fig. 3) in comparison to Control (interaction of treatment site and time, *P* ˂ 0.01). At the Bumetanide site, CVC was elevated at the end of the low‐intensity exercise and Recovery 1 in comparison to Control (both *P* ≤ 0.05) but similar during the other time periods (all *P* ≥ 0.07). Perfusion of BaCl_2_ resulted in similar levels of CVC compared to Control at Baseline and the end of the low intensity exercise (both *P* ≥ 0.13) but attenuated CVC at the remaining time periods (all *P* ˂ 0.05). ∆CVC from Control at the Ouabain site was similar during each exercise bout (all *P* ≥ 0.23; Fig. 4). Bumetanide administration resulted in greater ∆CVC from Control during both the moderate and high compared to the low‐intensity exercise bout (both *P* ˂ 0.05). At the BaCl_2_ site, ∆CVC from Control was greater during high compared to low and moderate intensity exercise (both *P* ˂ 0.05). CVC_max_ was similar at all treatment sites (*P* ≥ 0.16).

### Cardiovascular and temperature responses

Heart rate was elevated compared to Baseline values at the end of each exercise and recovery period (all *P* ˂ 0.01; Table 1) with the exception of Recovery 1 (*P* = 0.12). At the end of the moderate and high intensity exercise, as well as Recovery 2 and 3, heart rate was increased compared to their respective previous time periods (all *P* ˂ 0.01). Mean arterial pressure was only elevated at the end of the moderate and high‐intensity exercise bout compared to baseline (both *P* ˂ 0.05), and greater at the end of the high compared to moderate intensity exercise (*P* ˂ 0.01).

**Table 1:** Temperature and cardiovascular responses during baseline, exercise, and recovery periods

	Baseline	LOW	Recovery 1	MODERATE	Recovery 2	HIGH	Recovery 3
		(30% VO_2peak_)		(50% VO_2peak_)		(70% VO_2peak_)	
Esophageal temperature, °C	36.8 ± 0.2	37.2 ± 0.2^a^	37.0 ± 0.2^a^	37.6 ± 0.1^a^, ^b^	37.1 ± 0.2^a^, ^b^	38.2 ± 0.1^a^, ^b^	37.4 ± 0.2^a^, ^b^
Mean skin temperature, °C	31.4 ± 0.3	32.4 ± 0.4^a^	32.3 ± 0.3^a^	33.5 ± 0.3^a^, ^b^	32.9 ± 0.4^a^, ^b^	33.8 ± 0.3^a^	33.2 ± 0.4^a^, ^b^
Mean body temperature, °C	36.2 ± 0.1	36.8 ± 0.1^a^	36.6 ± 0.2^a^	37.2 ± 0.1^a^, ^b^	36.7 ± 0.2^a^, ^b^	37.7 ± 0.2^a^, ^b^	37.0 ± 0.2^a^, ^b^
Mean arterial pressure, mmHg	90 ± 3	97 ± 4	89 ± 3	101 ± 7^a^	89 ± 5	108 ± 8^a^, ^b^	86 ± 5
Heart rate, bpm	65 ± 7	95 ± 7^a^	69 ± 7	125 ± 9^a^, ^b^	79 ± 8^a^, ^b^	160 ± 7^a^, ^b^	89 ± 7^a^, ^b^

Presented values (*n* = 11) are mean ± 95% confidence interval. Esophageal, mean skin and mean body temperatures, as well as heart rate values represent an average of the final 5 min for the corresponding time period. Mean arterial pressure values represent an average of two measurements from the final 10 min for each corresponding time period. Baseline values represent 5 min prior to the first exercise bout. LOW/MODERATE/HIGH, Low/Moderate/High intensity exercise bout; VO_2peak_, peak oxygen consumption; bpm, beats per minute.

a Significantly different versus Baseline.

b Significantly different versus previous period (i.e., exercise vs. exercise; recovery vs. recovery) (all *P* ≤ 0.05).

Compared to Baseline values, esophageal, mean skin, and mean body temperatures were elevated during all exercise and recovery periods (all *P* ˂ 0.01; Table 1). Esophageal, mean skin, and mean body temperature were greater at the end of moderate and high‐intensity exercise, as well as Recovery 2 and 3 compared to the low and moderate intensity exercise, and Recovery 1 and 2, respectively (all *P* ≤ 0.05) (with the exception of mean skin temperature at the end of the high compared to the moderate intensity exercise bout [*P* = 0.06]).

## Discussion

We demonstrated that the Na^+^/K^+^‐ATPase, NKCC, and K^+^ channels are involved in the regulation of the sweating and cutaneous vasodilatory response during exercise, and their apparent contribution varied based on the intensity of exercise. Local sweat rate was unaffected during the low‐intensity exercise at all treatment sites. However, Na^+^/K^+^‐ATPase inhibition resulted in a marked attenuation in the sweating response during the moderate and high‐intensity exercise bouts, whereas NKCC and K^+^ channel inhibition attenuated sweat rate during the high‐intensity exercise bout only. With regard to the regulation of cutaneous vasculature, Na^+^/K^+^‐ATPase inhibition resulted in an augmented cutaneous vasodilatory response throughout the incremental intermittent exercise protocol. Moreover, NKCC inhibition augmented cutaneous vasodilatation during low‐intensity exercise only, whereas K^+^ channel inhibition attenuated the response in the moderate and high‐intensity exercise bouts.

### Sweating response

In line with previous findings, we demonstrated an attenuation in sweat rate with ouabain administration (Sato and Dobson 1969; Sato et al. 1969; Louie et al. 2016), reiterating the role of the Na^+^/K^+^‐ATPase in regulating sweating during exercise. Although we previously reported a role for Na^+^/K^+^‐ATPase during exercise, this was examined under moderate intensity exercise only (Louie et al. 2016). In this study, we show that this response is only evidenced at exercise intensities of moderate or higher levels, while the role of Na^+^/K^+^‐ATPase in the regulation of sweating is absent during low‐intensity exercise. For the first time, we show that the NKCC and K^+^ channels play an important role in the regulation of local sweating in humans during exercise. By inhibiting these transporters, we observed marked attenuations in the sweating response when compared to the Control site, but their influence is limited to exercise intensities that result in higher rates of metabolic heat production. These findings build upon previous findings that demonstrated the inhibition of the NKCC and K^+^ channels (via bumetanide and Ba^2+^, respectively) caused a rapid cessation in methacholine‐induced sweat rate in isolated sweat glands in vitro (Sato and Sato 1987). Given we did not observe a complete suppression in sweating with NKCC inhibition, our findings support that other transporters located on the sweat gland basolateral membrane likely contribute to the influx of Cl^−^ such as the Cl^−^/HCO_3_ exchanger (Sato and Sato 1987; Wilson and Metzler‐Wilson 2015).

### Cutaneous vascular response

Consistent with our previous study (Louie et al. 2016), Na^+^/K^+^‐ATPase inhibition resulted in augmented levels of CVC compared to Control during the Baseline and all recovery periods; the latter response occurring irrespective of the greater exercise‐induced rates of metabolic heat production. By simultaneously inhibiting nitric oxide synthase, we had previously determined that this augmented cutaneous vasodilatory response was nitric oxide dependent (Louie et al. 2016). However, we also showed that CVC was similar at the Na^+^/K^+^‐ATPase inhibited and Control sites as assessed during two successive bouts of moderate (~50% VO_2peak_) intensity exercise (Louie et al. 2016). On the other hand, in this study, we observed an augmented CVC response during the three successive exercise bouts performed at increasing intensities. These contrasting findings may be explained by the warm environment (35°C) employed in the previous study, resulting in greater levels of mean skin temperature (Louie et al. 2016) compared to those measured in this study. It is well known that the underlying mechanisms regulating cutaneous vasodilatation in response to elevations in local skin temperature alone can differ compared to those observed during whole‐body heat stress and exercise (Johnson et al. 2014). However, it is unclear to what extent that differences in skin temperature between exercise in thermoneutral conditions and in the heat can explain the discrepancy in the mechanisms underpinning cutaneous vasodilatation.

We observed that inhibition of the NKCC with bumetanide resulted in greater levels of CVC compared to Control during the low‐intensity exercise bout. This elevated CVC response has been demonstrated in rats, using both isolated vasculature in vitro and intravenous injection in vivo, in which vasodilatory responses were seen following the administration of the NKCC inhibitor, furosemide (Barthelmebs et al. 1994). Liguori et al. (1999), using human endothelial cells in vitro as well as intravenous infusion in humans in vivo, demonstrated that exposure to furosemide resulted in greater release of prostacyclin (PGI_2_), a potent vasodilator. However, we recently demonstrated that nonselective inhibition of cyclooxygenase, an enzyme responsible for the production of prostaglandins including PGI_2_, did not have any effect on the cutaneous vascular response during both moderate‐ and high‐intensity exercise in the heat (Fujii et al. 2014). Taken together, the augmented CVC response seen in this study with bumetanide administration may not be related to prostaglandin synthesis per se, but may be related to other vasodilators such as endothelial kinins (e.g., bradykinin) and/or nitric oxide that have been shown to increase in production with furosemide in bovine aortic endothelial cells (Wiemer et al. 1994).

The attenuated CVC response during exercise with BaCl_2_ administration in this study builds upon recent observations demonstrating a role for K^+^ channels in the regulation of cutaneous vasculature tone. Activation of K^+^ channels located on endothelial cells results in hyperpolarization which can travel to vascular smooth muscle cells (VSMCs) via the gap junctions. Additionally, activation of K^+^ channels on the VSMC itself can result in hyperpolarization. The hyperpolarization of VSMCs induces a relaxation of smooth muscle, thereby resulting in vasodilatation (Feletou and Vanhoutte 2009; Edwards et al. 2010). However, Brunt et al. (2013) observed that during whole‐body passive heating, K_Ca_ blockade did not attenuate the cutaneous vasodilatory response. Based on the study by Brunt et al. (2013) and the present findings, K^+^ channels other than K_Ca_ channels may play a role in the cutaneous vasodilatation during whole‐body heating, such as K_ATP_ (Hojs et al. 2009) and/or K_V_ (Ferrer et al. 1999; Gupta et al. 2008) channels.

### Exercise intensity‐dependent contributions

As previously discussed, there exist a number of factors that may affect the sweating and cutaneous vasodilatory response which increase in production or activation with the level of exercise intensity, such as aldosterone and vasopressin (Montain et al. 1997), heat‐shock proteins (i.e., HSP70) (Milne and Noble 2002), endothelin‐1 (Maeda et al. 1994), and oxidative stress (Goto et al. 2003). For instance, oxidative stress is known to influence the function of various membrane transport proteins including the Na^+^/K^+^‐ATPase, NKCC, and K^+^ channel (Elliott and Schilling 1992; Elliott and Koliwad 1995; Liu and Gutterman 2002). Given that previous work demonstrating elevated levels of an oxidative stress marker, malondialdehyde, following 30 min of high (i.e., 75% VO_2peak_) but not moderate (i.e., 50% VO_2peak_) intensity exercise (Goto et al. 2003), we had previously conducted a study to assess the effects of ascorbate, an antioxidant, on the cutaneous vasodilatory response during exercise. We observed that local administration of ascorbate resulted in elevated levels of CVC compared to a control site during high‐intensity exercise (71 ± 8% VO_2peak_); a response which was not seen during moderate intensity exercise (52 ± 6% VO_2peak_) (Meade et al. 2015). Further scrutiny is warranted to evaluate if the exercise intensity‐dependent increases in oxidative stress, or other factors such as those listed previously, can explain the varying contributions of the transmembrane proteins to the sweating and cutaneous vasodilatory responses observed in this study.

### Limitations

A primary limitation of the intradermal microdialysis technique employed in this study is that we can only administer the inhibitory agents in a nonspecific fashion. For example, it is not currently possible to target specifically the basolateral, luminal, or ductal membrane of the sweat gland in humans in vivo without affecting the other membranes. Similarly, pertaining to the cutaneous vasculature, the Na^+^/K^+^‐ATPase, NKCC, and K^+^ channels were likely inhibited on both the endothelial and vascular smooth muscle cells. Additionally, the influence of the pharmacological agents administered on surrounding cells (e.g., keratinocytes, antigen‐presenting cells, mast cells, etc.) cannot be directly determined. Thus, the sweating and cutaneous vasodilatory data must be interpreted as the response to nonspecific inhibition.

Greater levels of exercise intensity and therefore rates of metabolic heat production are accompanied by elevated thermal drive leading to an increase thermoeffector activity and thus rate of heat loss (Gagnon et al. 2013; Kenny and Jay 2013). A potential limitation of the experimental design is that the successive exercise bouts can lead to progressively greater increases in thermal drive, which may have been more pronounced than if we had opted to have the participants perform each exercise intensity on separate days. However, the microdialysis technique is limited in the sense that performing a multiple day experiment may not be feasible since the insertions and equipment would have to be placed at the exact same locations (i.e., to avoid regional variation). The responses observed in this study may have been influenced by increases in cumulative heat storage with each successive exercise bout and therefore thermal drive, a response that would not be seen with the separate day protocol. Taken together, our findings should be interpreted carefully and future studies employing non‐exercise passive heating models are warranted to determine if the observations made in this study are the results of changes in the thermoeffector activity (i.e., magnitude of sweat rate and cutaneous vascular conductance) associated with a greater core temperature, and not exercise intensity per se.

### Perspectives

The findings of this study further our knowledge pertaining to the underlying mechanisms that regulate cutaneous vasodilatation and sweating during exercise. These data can provide insight for future research determining through which specific end‐organ mechanisms (e.g., Na^+^/K^+^‐ATPase, NKCC, and K^+^ channels) that upstream modulators, such as nitric oxide and cyclooxygenase, modulate their influence on the heat loss responses. Moreover, for the first time we demonstrated a key role for K^+^ channels in regulating cutaneous vasodilatation during exercise using nonspecific K^+^ channel inhibition (i.e., BaCl_2_ administration). As previously mentioned, given that inhibitors are available for safe use in humans to study the specific K^+^ channel subtypes (Ferrer et al. 1999; Gupta et al. 2008; Hojs et al. 2009; Brunt et al. 2013), it remains to be determined which subtype(s) can explain the cutaneous vasodilatory and sweating responses seen in this study.

## Conclusion

We demonstrated the roles of the Na^+^/K^+^‐ATPase and, for the first time, the NKCC and K^+^ channels in regulating the sweating and cutaneous vascular response during exercise. Furthermore, their contributions to these responses seemed to differ as a function of exercise intensity (i.e., 30%, 50%, and 70% VO_2peak_) and therefore rates of metabolic heat production.

## Conflict of Interest

None declared.
